# Well-being in parents of children with cancer: illness perceptions’ mediating role for hope and social support

**DOI:** 10.3389/fpsyg.2024.1206520

**Published:** 2024-07-03

**Authors:** Irit Schwartz-Attias, Tamar Krulik, Tammie Ronen

**Affiliations:** ^1^Meir Academic Nursing School, Meir Medical Center, Clalit Health Services, Kfar Saba, Israel; ^2^Department of Nursing, Faculty of Medical & Health Sciences, Steyer School of Health Professions, Tel Aviv University, Tel Aviv, Israel; ^3^Faculty of Social Sciences, Tel-Aviv University, Tel Aviv, Israel

**Keywords:** social support, hope, childhood cancer, parents, subjective well-being

## Abstract

**Introduction:**

Parents of children with cancer may experience enormous physical and emotional pressures. During such times, perception of the situation can be important in mediating the link between one’s basic resources (mainly cognitive and social) and the well-being one attains as an emotional and cognitive response. This study aimed to explore the role of illness impact perceptions in mediating the link between hope, social support and subjective well-being in parents of children with cancer.

**Methods:**

The cross-sectional study included 108 parents of children aged 7–18 diagnosed with cancer at least 6 months prior to the study. The parents completed one questionnaire comprising five instruments: perceived impact of illness, hope, social support, positive and negative affect (the emotional component of well-being) and life satisfaction (the cognitive component of well-being). Descriptive statistics and Pearson correlations were conducted for all study variables. SEM analysis was performed to examine the study’s theoretical model.

**Results:**

The current sample included 108 parents of children with cancer recruited from two pediatric hematology-oncology wards in two different hospitals in central Israel. Most participants were mothers (70.4%), and the mean age was 44.46. The main results indicated that hope and social support correlated negatively with perceptions of the illness’ impact. Illness impact perceptions mediated the relations between hope, social support and positive emotions, which means that when the parents perceived their child’s illness as less impactful on the family, they experienced higher levels of emotional well-being.

**Conclusion:**

A parent with social support resources and higher levels of hope experiences higher levels of positive perceptions regarding their child’s illness. Higher levels of positive perceptions allow the parent to express more positive than negative emotions, thus maintaining a more optimal level of subjective emotional well-being. The findings offer implications for healthcare teams to enhance sensitivity to parents’ needs and to help parents attain more resources, positive perceptions, and well-being.

## Introduction

Pediatric cancer is a chronic disease that generates enormous physical and emotional pressures for the patient, the family, and the social environment (Modanloo et al., 2019). Every year, approximately 300,000 children aged 0 to 19 are diagnosed with cancer worldwide (Steliarova-Foucher et al., 2017). Despite progress in medical care, cancer is still perceived as extremely life-threatening by pediatric patients and their families. Therefore, parents’ perceptions are a key element in their coping style (Bally et al., 2014). Parents must cope with and adapt to the new stressful situation (Lazarus and Folkman, 1984; Bemis et al., 2015). Thus, their belief that they can cope, as well as their ability to actually cope with stress, is critical in maintaining children’s and parents’ well-being (Bally et al., 2014).

When parents perceive their child’s illness as more severe, they will experience more psychological distress (Juth et al., 2015; Bilani et al., 2019). Beck (2019) stated that it is not the problem but the interpretation of the problem that influences people’s coping. Perceptions of illness are essential for the optimal care of ill children. Primary caregivers’ subjective perceptions of illness severity and pathology have been shown to differ from objective indices of patients’ actual illness (Broadbent et al., 2006). Therefore, better perceptions of their child’s illness may be of great importance to the ability to maintain their well-being. Previous studies have revealed links between perceptions of illness and compliance, recovery, self-control, and quality of life (Salvador et al., 2015). Is there a way to change perceptions?

Perception has been found to potentially be linked to basic resources. Hobfoll’s (2004) “conservation of resources” model asserted that when people lose one resource, they act in search of other resources to reach balance; hence, when facing pediatric cancer, the loss of their child’s health may lead parents to seek alternative resources for coping. Coping mechanisms are necessary to achieve higher levels of functioning (Lazarus and Folkman, 1984). Such mechanisms offer hope and social support as crucial coping resources in human life in general and at times of stress in particular. Parents who possess hope and social support resources have been found to better manage their perceptions of illness, which increases their well-being (Lazarus and Folkman, 1984).

Based on this, it can be assumed that parents’ more favorable illness perceptions derive from two basic resources: the individual resource of hope and the environmental resource of social support.

Personal hope is an internal mechanism. Snyder (2000) defined hope as a goal-directed construct comprising three components. The first component consists of having a goal that is valuable but uncertain. The second is pathway thinking, which refers to one’s perception of oneself as capable of producing multiple workable routes leading to one’s goal, despite possible barriers. The third is agency thinking, which refers to the perception that one is capable of initiating and sustaining movement along these pathways to achieve one’s goal. Research on hope among parents of children with cancer has revealed that this resource strengthens parents and helps them cope with the difficult situations associated with the illness and its treatment (Eche et al., 2020). A qualitative study conducted among 20 parents of children with cancer revealed that the establishment of hope had a profound impact on parents at both the emotional and cognitive levels. This hope was found to improve the parents’ overall mood, foster greater acceptance of the reality they were facing, and empower them to effectively care for themselves and their children (Williams et al., 2023). However, the possible links between hope and positive emotions have not yet been sufficiently investigated. In this study, we propose that hope will enable more favorable perceptions of the child’s illness, which may enable higher parental well-being.

The second resource that has been studied is social support, which is considered an environmental mechanism. Cohen et al. (1985) defined social support as a situation in which one feels belonging to a group, which encompasses mutual relationships, commitment, and responsibility. Positive social psychology scholars have consistently highlighted the social context – specifically relationships – as the most important contextual factor affecting individual well-being and enabling the individual to cope better with stressful life events and live a healthy life (Lomas, 2015). Likewise, a negative relationship has been shown between social support and mental distress (Hildenbrand et al., 2011; Harper et al., 2016; Boyden et al., 2020). Social support encompasses significant resilience and a buffering factor against the negative implications of stress (Schwartz-Attias et al., 2023). Stronger social support has been correlated with a higher level of well-being, which is reflected in life satisfaction, the cognitive component of well-being, and positive emotions, reflecting the emotional component of well-being (Gise and Cohen, 2022; Melguizo-Garín et al., 2022), as well as lower levels of stress, hopelessness, and depression (Makhoul Khoury et al., 2018). Little is known about the relation between social support and hope among parents of children with cancer. However, evidence from other populations has shown that both resources are vital (Xiang et al., 2020).

We, therefore, expect illness perception to mediate the link between social support and hope and subjective well-being.

Well-being is among the many concepts employed in relation to the human ability to conduct a full, rich life, such as happiness, life satisfaction, and flourishing. The general well-being concept includes both cognitive and emotional dimensions. In this study, we examine parents’ life satisfaction, which relates to the cognitive elements, and their positive and negative affect, which relate to the emotional component of well-being. Inasmuch as both are internal components, Diener (1984) has asserted that only individuals can attest to their own subjective well-being.

Satisfaction with life refers to the degree to which one subjectively appreciates one’s life, comparing expectations and goals with the ability to achieve these goals (Diener, 1984). Bandura (2006) underscored that for people to be satisfied with life, they must focus not on the environment but on the actions they take to realize their goals, as ultimately, these actions will improve their lives. A comparative study revealed lower life satisfaction among parents of children with cancer compared to parents of healthy children (Baran et al., 2020).

While life satisfaction encompasses the cognitive components of well-being, positive emotion encompasses the emotional components of well-being, with both positive and negative affects necessary for human survival (Fredrickson, 2013). Fredrickson (2013) viewed positive emotions as a type of immunizing mechanism that allows the human body to not only reduce the impact of negative emotions but also actively facilitate the sense of control that increases one’s ability to cope with stressful situations. Lazarus and Folkman (1984) explored the role of positive influences during stressful situations. They asserted that, despite the negative emotions that dominate during times of crisis, positive emotions can provide a psychological “break” from the stress, thereby providing support and enabling people to continue their coping attempts and renew their resources and depleted assets caused by the situation. Pressman and Cohen (2005) demonstrated that positive emotions exist during situations of crisis and illness. However, the relations between positive and negative emotions have not yet been sufficiently investigated among parents of children with cancer.

The proportion of positive affect compared to negative affect is termed a “positivity ratio” (Fredrickson, 2013). When positive emotions (e.g., enthusiasm, pride, determination) outweigh negative emotions (e.g., fear, frustration, guilt), then one can be assessed as having a high positivity ratio – a high level of the emotional dimension of subjective well-being (Fredrickson, 2013). The conceptualization of positivity ratio derives from the idea that positive and negative affect operate as independent bipolar constructs, so that the existence of one does not necessarily point to a lack of the other (Watson et al., 1988). Yet, the psychological impacts of unpleasant phenomena generally tend to outweigh those of pleasant phenomena. Hence, a single bad event usually has a greater impact than a comparable good event (Sparks and Baumeister, 2008). Consequently, a larger quantity of positive emotional experiences is necessary to counteract the impact of negative experiences. Therefore, we sought to explore parents’ positivity ratio as an important measure of their well-being when facing their child’s cancer.

In sum, the current study examines parents’ perceptions about the impact of their child’s illness as potentially mediating the links between hope, social support and their own subjective well-being. The study integrates cognitive-behavioral theory, stress-and-coping studies, and positive psychology. The cognitive-behavioral approach upholds that it is not necessarily an objective problem that leads to subjective distress but rather one’s perception of the problem (Ronen and Seeman, 2007). Thus, the current study focused on parents’ subjective perceptions of the illness’s impact and their own well-being and included three hypotheses:

1. Parents’ illness-impact perceptions will significantly negatively correlate with parents’ subjective well-being. Namely, a less severe perceived impact of the child’s illness will correlate with parents’ higher positivity ratio and a higher life satisfaction level.
2. Illness impact perceptions will mediate the correlation between parent’s hope and subjective well-being.
3. Illness impact perceptions will mediate the correlation between parents’ social support and subjective well-being.

## Method

### Design

This study was a descriptive cross-sectional survey that examined the illness perception, hope, social support and subjective well-being among parents of children with cancer.

### Sample

The current sample included 108 (94% Jewish, 6% Muslim) parents of children with cancer recruited from two pediatric hematology-oncology wards in two different hospitals in central Israel with 60 beds altogether. The inclusion criteria were as follows: (a) parents of children aged 7–18; (b) parents of children diagnosed with cancer at least 6 months prior to the study and no more than one year after the termination of treatments; (c) ability to communicate in Hebrew; and (d) parental informed consent to participate in the study. In each hospital ward, data were collected from the child’s computerized records to assess the parents who met the criteria for inclusion in the study. Initial contact was made with the parents to explain the aims of the study. Parents who agreed to participate in the study defined themselves as primary caregivers. At the time of data collection, some patients were still in active treatment, some were in maintenance (e.g., with leukemia), and some were off treatment but for no more than one year. Characteristics of the parents and children are shown in Table 1.

**Table 1:** Parents’ and children’s characteristics.

Characteristics	Categories	Parents, *n* (%)	Children, *n* (%)
Sex	Female	76 (70.4)	44 (40.7)
	Male	32 (29.6)	64 (59.3)
Age in years	Range	30–62	7–18
	*M*	44.46	13.11
	SD	6.10	3.60
Parent’s marital status	Married	89 (82.4)	—
	Other	19 (17.6)	—
Parent’s religion	Jewish	101 (93.5)	__
	Muslim	7 (6.5)	__
Parent’s level of religiosity	Secular	37 (34.3)	__
	Traditional	53 (49)	__
	Orthodox	18 (16.7)	__
Parents worked before child’s diagnosis	Yes	94 (87)	__
	No	14 (13)	__
Parent’s level of education	High school	32 (29.63)	__
	Professional	28 (25.93)	__
	Bachelor’s degree	31 (28.70)	__
	Master’s degree	12 (11.11)	__
	PhD	2 (1.85)	__
	Other	3 (2.78)	__
Child’s treatment phase	Active treatment phase	—	32 (29.6)
	1 month since treatment ended	—	11 (10.2)
	2 months since treatment ended	—	11 (10.2)
	3–12 months since treatment ended	—	54 (50.0)
Child’s diagnosis	Acute lymphoblastic Leukemia	—	42 (39)
	Bone cancer	—	25 (23)
	Lymphoma	—	17 (16)
	Acute myeloid leukemia	—	9 (8)
	Rhabdomyosarcoma	—	3 (3)
	Neuroblastoma	—	3 (3)
	Wilms tumor	—	2 (2)
	Rare oncology disease	—	7 (6)

### Measures

All five research instruments administered to parents had previously undergone back-translation from English into Hebrew and into English again, demonstrating acceptable reliability. The Hebrew version of these instruments have been used across many studies with good reliability (e.g., Ronen et al., 2016; Orkibi et al., 2018; Bukchin-Peles and Ronen, 2022).

#### Perceived impact of illness

The 8-item Brief Illness Perception Questionnaire (Broadbent et al., 2006) was based on the Revised Illness Perception Questionnaire (Moss-Morris et al., 2002). For the present study, we adapted this self-report to parents and utilized only the five items assessing parents’ perceptions about the degree to which the child’s illness affected the parent (i.e., How much does your child’s illness affect your life? How long do you expect your child’s illness will last? To what extent do you believe the treatments your child receives will help them recover? To what extent are you concerned about your child’s illness? To what extent does your child’s illness affect you emotionally, for example, makes you angry, afraid, depressed?) and one item assessing parents’ perceptions about the degree to which the illness affects the child (To what extent does your child feel the symptoms caused by their illness?). The parents rated these six items from 0 to 10, where higher scores indicated more negative impact. Cronbach alpha was 0.88 in Broadbent et al. (2006) and 0.65 for the six items in the present study.

#### Hope

The 6-item Adult State Hope Scale (Snyder, 2000) assessed parents’ perceptions of hope in the past week (e.g., Agency: “At the present time, I am energetically pursuing my goals;” Pathway: “There are many ways around any problem that I am facing now”), rated on an 8-point Likert scale ranging from “strongly disagree” (1) to “very strongly agree” (8). Higher total scores indicated higher levels of hope. Cronbach alpha was 0.95 in Snyder (2000) and 0.90 in the present study, indicating good reliability.

#### Social support

This 12-item brief social support questionnaire assessed parents’ perceived availability of social resources and was derived from the original 40-item Interpersonal Support Evaluation List (ISEL)–General Population (Cohen et al., 1985). The parents rated items (e.g., “I know who I can turn to when I have a problem with my family”) on a 4-point Likert scale ranging from “very untrue” (0) to “very true” (3). Higher scores indicated higher perceived social support. Cronbach alpha was 0.86 in Cohen et al. (1985) and 0.89 in the present study, indicating good reliability.

#### Positivity ratio: emotional dimension of subjective well-being

Positivity ratio was calculated as the ratio between positive and negative emotions measured on the 20-item Positive and Negative Affect Scale (Watson et al., 1988). A high positivity ratio score indicated that the parents experienced more positive than negative emotions (Fredrickson, 2013). This scale included 10 positive emotions (e.g., excited, contented) and 10 negative emotions (e.g., agitated, sad), rated on a 5-point Likert scale ranging from “very little” (1) to “very much” (5). Higher scores indicated a stronger impact of that type of emotion (positive or negative). Cronbach alphas were 0.88 for positive emotions and 0.87 for negative emotions in Watson et al. (1988) and were 0.71 for positive emotions and 0.86 for negative emotions in the present study.

#### Life satisfaction: cognitive dimension of subjective well-being

The 7-item Life Satisfaction Scale (Huebner, 1991; translation to Hebrew in Ronen and Seeman, 2007) assessed parents’ overall life satisfaction, rated on a 4-point Likert scale ranging from “never relevant” (1) to “almost always relevant” (4). Higher scores indicated larger degrees of life satisfaction. Cronbach alpha was 0.82 in Ronen and Seeman (2007) and 0.81 in the present study, indicating good reliability.

#### Demographic information and child’s illness characteristics

Family members’ sociodemographic data and children’s medical data were collected.

### Procedure

Approval to conduct the study was received from the ethics committee at each of the two hospitals with pediatric cancer units (# 0178-13-RMC, # 0611-14-TLV, respectively). Next, parents who met the study criteria were approached in writing to request their participation. Of the 125 families approached, 108 gave written consent and completed the questionnaires. Completion of the questionnaires by one parent per family took approximately 45 min.

### Data analysis

We conducted descriptive statistics and Pearson correlations for all study variables using SAS software, 9.4 version. We performed structural equation modeling (SEM) to examine the study’s theoretical model using the Mplus Version 8.6 (Muthén and Muthén, 1998–2017). We specified hope, social support, illness impact perceptions, and well-being as latent constructs in the model. We measured each using the accepted parceling approach (Bandalos, 2002), with three indicators calculated as random thirds of the scale items.

We modeled parents’ positivity ratio and sociodemographic variables as observable variables. We used the Mplus MLR estimator, which enables a type of maximum likelihood estimation with robust standard errors and chi-square calculation in the presence of deviation of data from normality (Little and Rubin, 2003). We assessed the model’s fit to the data using chi-square, with a low value indicating a good fit. The data underlying the findings are available in this article.

## Results

### Descriptive statistics for study variables

Analysis of variance comparing age, sex, severity of illness, and stage of treatment did not show any significant differences between the groups, we, therefore, related to it as one group. As shown in Table 2, the mean scores of the questionnaires indicated the parents’ high levels of both hope (6.5 on a 1–8 scale) and social support (2.31 on a 0–3 scale). With regard to subjective well-being, the parents showed high means for both life satisfaction (2.95 on a 1–4 scale) and positivity ratio (1.82 for positive to negative emotions). With regard to illness-impact perceptions, the parents’ mean score of 5.62 (on a 0–10 scale) expressed a moderate level of concern about the impact of their child’s illness.

**Table 2:** Descriptive statistics for study variables.

Variables	Minimum-maximum	Mean (SD)
Parent’s illness impact perceptions (range 0–10)	0.83–9.17	5.62 (1.49)
Parent’s hope (range 1–8)	3.17–8	6.5 (1.07)
Parent’s social support (range 0–3)	0.83–3	2.31 (0.58)
Parent’s positivity ratio	0.70–4.27	1.82 (0.77)
Parent’s life satisfaction (range 1–4)	1.57–4	2.95 (0.54)

### Correlations among study variables

As seen in Table 3, moderately high significant correlations emerged among all the study variables. In line with the first hypothesis, a perception of lower illness impact attributed by parents to their child’s illness was correlated with parents’ higher life satisfaction (*r* = −0.39*; p* < 0.001) and higher positivity ratio (*r* = −0.55*; p* < 0.001). Thus, the first hypothesis was supported.

**Table 3:** Intercorrelations among parents’ study variables.

Parents’ study variables	Illness-impact perception	Hope	Social support	Positivity ratio
Hope	−0.35^**^			
Social support	−0.37^***^	0.41^***^		
Positivity ratio	−0.55^***^	0.47^***^	0.52^***^	
Life satisfaction	−0.39^***^	0.54^***^	0.53^***^	0.53^***^

^***^p < 0.001; ^**^p < 0.01.

### Correlations between study variables and socio-demographic variables

As shown in Table 4, significant correlations emerged between some of the parents’ demographic variables and study variables. Higher levels of social support (*r* = 0.23, *p* = 0.02), hope (*r* = 0.20, *p* = 0.04), positive emotions (*r* = 0.25, *p* < 0.001), and life satisfaction (*r* = 0.26, *p* = 0.001) were found among parents who worked before the child was diagnosed. Married parents reported higher levels of social support (*r* = 0.21, *p* = 0.02), positive emotions (*r* = 0.20, *p* = 0.04), and life satisfaction (*r* = 0.21, *p* = 0.03) compared to non-married parents. More highly educated parents showed lower illness impact perceptions (*r* = −0.22, *p* = 0.02) and higher levels of life satisfaction (*r* = 0.25, *p* < 0.01). The parents of children diagnosed with acute lymphoblastic leukemia (ALL) reported higher levels of social support (*r* = 0.28, *p* < 0.01) and positive emotions (*r* = 0.31, *p* = 0.001) and lower illness impact perceptions (*r* = 0.21, *p* = 0.03) compared to other diagnoses.

**Table 4:** Pearson correlations among parents’ sociodemographic variables and study variables.

Study variable/sociodemographic variables	Illness-impact perception	Hope	Social support	Positivity ratio	Life satisfaction
Jewish	−0.21^*^	0.05	0.15	0.19^*^	0.08
Secular	−0.15	0.12	0.08	0.09	−0.04
Marriage status	−0.15	0.07	0.21^*^	0.20^*^	. ^*^21
Worked before child diagnosed	−0.17	0.20^*^	0.23^*^	0.25^**^	0.26^**^
Level of education	−0.22^*^	0.10	0.05	0.02	0.25^**^
Child’s all diagnosis	−0.21^*^	0.10	0.28^**^	0.31^***^	0.17

^***^p ≤ 0.001; ^**^p < 0.01; ^*^p ≤ 0.05.

### Structural equation modeling

The SEM analysis used to examine the remaining hypotheses showed a good fit between the research hypotheses and measurement model: χ^2^ (48) = 65.10, *p* = 0.051. Since age, sex, severity of illness and stage of treatment did not contribute significantly to positivity ratio, we did not include them in the model. To control for the demographic variables that showed significant correlations with at least one independent or dependent study variable, we added the following to the model as control variables: parents’ religion, marital status, work before diagnosis, level of education and the child’s diagnosis (ALL vs. other). The research model showed a good fit, with no significant chi-square coefficient: χ^2^ (104) = 117.15, *p* = 0.178. Next, we omitted the insignificant paths from the background variables to the study variables and re-ran the model, which again showed a good fit to the data: χ^2^ (103) = 123.91, *p* = 0.079.

**Figure 1:**
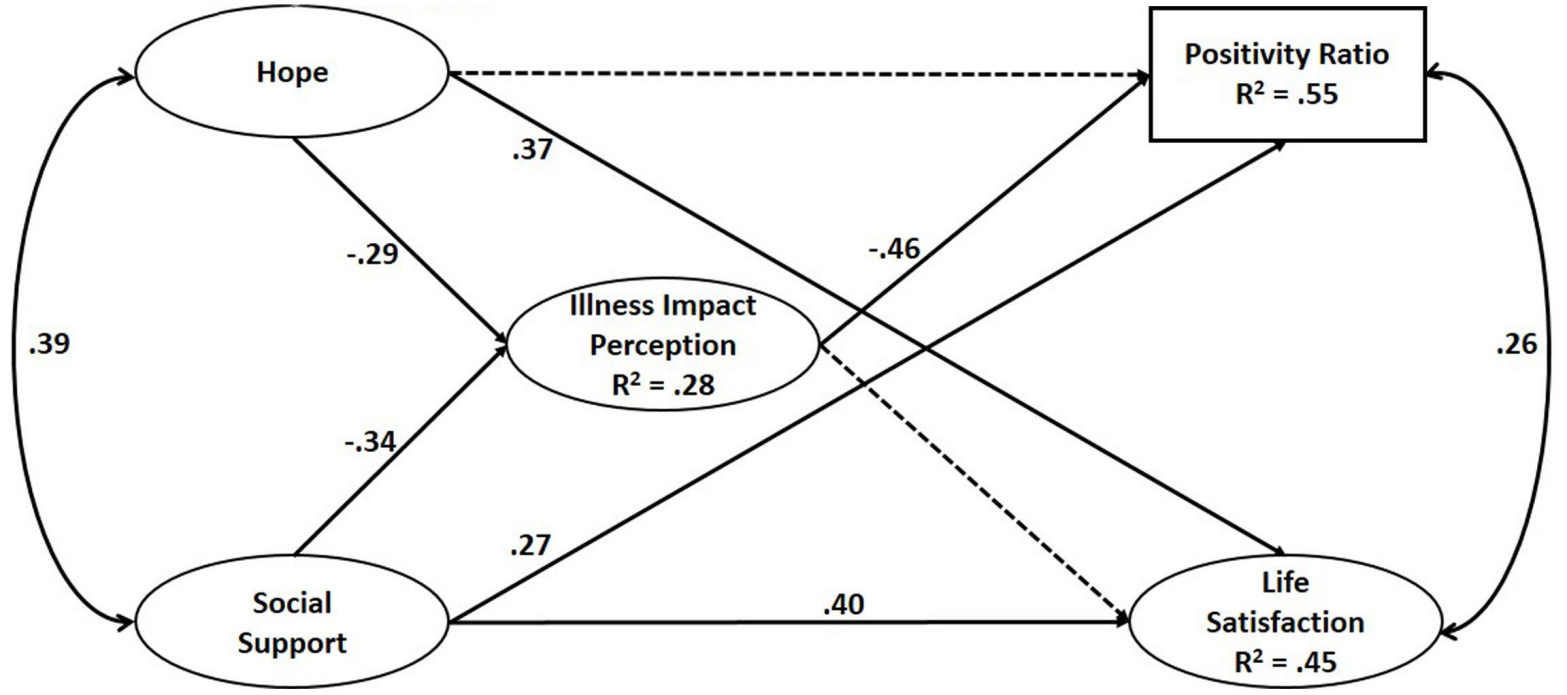
Structural equation modeling of parent’s variables: hope, social support, illness impact perception, and subjective well being (*N* = 108). The solid lines indicate paths statistically significant at *p* < 0.05. The dotted line indicate non significant paths.

As shown in Figure 1, the SEM indicated significant direct effects between hope and life satisfaction (β = 0.37, *p* < 0.05), between social support and life satisfaction (β = 0.40, *p* < 0.05), and between social support and positivity ratio (β = 0.27, *p* < 0.05). Negative direct effects also emerged between the independent variables (hope and social support) and illness impact perceptions as well as between illness impact perceptions and positivity ratio (β = −0.46, *p* < 0.05). Regarding the second hypothesis, illness impact perceptions mediated only the effect between hope and positivity ratio (β = 14, *p* = 0.03). The indirect effect between hope and life satisfaction was not significant (β = 0.03, *p* = 0.47). This partially confirmed the second hypothesis.

The findings also lent partial support to the hypothesis regarding the mediating role of illness perceptions between social support and the two dimensions of well-being. The indirect effect between social support and positivity ratio was significant (β = 0.15, *p* = 0.016). However, no mediation emerged for the effect between social support and life satisfaction (β = 0.03, *p* = 0.460).

## Discussion

This study provided a look at parents of children aged 7–18 with pediatric cancer, spotlighting the ability of parents to attain a high level of well-being, both cognitive (being satisfied with their lives) and emotional (experiencing more positive than negative emotions), despite their child’s illness. The findings revealed the importance of illness perceptions and personal and environmental resources as coping mechanisms to help the parents maintain their well-being. The parents’ hope and social support and fewer negative illness perceptions were important resources that contributed to their well-being. Thus, the main contribution of the present study was its focus on the way existing resources can contribute to the perception of the illness’ impact. Nevertheless, the study also revealed the mediating role of the illness-impact perception on the parents’ ability to attain high levels of well-being despite the illness.

### Parents’ subjective well-being

Well-being has become a research focus over recent years, emphasizing that life includes various crises and challenges. Attaining well-being points to the ability to become resilient and cope despite obstacles. This study’s main outcome was that parents of ill children experience emotional well-being, with more positive than negative emotions at a ratio of 1.82. Furthermore, they also express cognitive well-being, with a high degree of life satisfaction. These high levels of subjective well-being, even when facing an extremely stressful life event, like a child’s life-threatening illness, hold optimistic implications for these parents’ coping. This finding supports similar studies’ findings that Muslim and Jewish parents of children with cancer experience more positive than negative emotions (Abu-Raiya et al., 2015; Schwartz-Attias et al., 2023).

Based on Fredrickson’s (2013) theory, more positive emotions lead people to adjust better to challenging situations and even to flourish. A possible explanation for parents’ resilient well-being may be related to Headey and Wearing’s (1992) claim that human beings have a fixed emotional point to which they are drawn back after any change in circumstances. The resilient well-being rates of parents facing their child’s illness may offer important insights to health professionals, suggesting that these parents can be treated as people who may, over time, learn to make the most of their challenging situation, instead of people who deserve pity and sorrow. In the past, most studies have focused on the negative aspects of illness and the need to cope. However, the present study focused on the ability to achieve well-being. Such a focus can aid helping professionals in their daily work with parents of ill children to achieve higher levels of well-being, changing the focus from sickness to well-being.

### Parents’ illness-impact perceptions influencing subjective well-being

The current study revealed the significant impact of parents’ illness-impact perceptions on their cognitive and emotional well-being. Bandura’s theory (1997), which emphasizes the role of expectancies and perceptions in shaping behavior, provides a possible explanation for these findings. Parents’ perceptions are influenced by hope and play a crucial role in their belief in their capacity to effect change and take positive actions. As a result, it is reasonable to expect that parents with more positive perceptions would experience better overall well-being.

This study’s intercorrelation findings revealed that when parents perceived their child’s illness as having a lesser impact on the family, they tended to experience enhanced emotional well-being, reflected in a higher ratio of positive to negative emotions. Moreover, these positive perceptions also correlated with greater cognitive satisfaction with their lives.

The study’s in-depth path analysis also demonstrated that illness perceptions act as a mediator between hope, social support, and parents’ emotions. This implies that when parents perceive their child’s illness as having a lesser impact on the family, they are more likely to experience heightened levels of positive emotions. In other words, hope and social support indirectly influence parents’ emotional well-being by shaping their perceptions of the illness’s impact.

The study’s outcomes for families of young children with illnesses align with previous research on parents of adolescents and young adults (aged 12–24) undergoing active cancer treatment (Juth et al., 2015). This consistency suggests that the role of perceptions in influencing emotions and well-being is applicable across different age groups and stages of illness. Therefore, this study underscores the vital role of perceptions in shaping parental well-being. It also highlights the importance of fostering positive perceptions through interventions that promote hope, social support, and a constructive outlook on the impact of a child’s illness on the family. By addressing and enhancing positive perceptions, such interventions can have meaningful implications for improving the emotional and cognitive well-being of parents facing the challenges of having a young child with an illness.

### Parents’ personal resource: hope

Hope was shown to be a crucial component in increasing both emotional and cognitive well-being experiences in the parents of ill children. The intercorrelations indicated that hope was correlated with life satisfaction (cognitive experience) and positivity ratio (emotional experience). The path analysis results indicated that hope showed a significant direct effect only on life satisfaction and not the positivity ratio. In line with the current model, an indirect effect emerged between hope and positivity ratio that went beyond the parents’ illness-impact perceptions, indicating that hope has the potential to increase parents’ positive emotions through their perceptions. Furthermore, hope can be increased by presenting parents with successful treatments and exposing them to the positive effects of the therapy.

According to Snyder (2000), people who score high on hope believe that they can adapt to potential difficulties and losses, entering into positive cognitive dialogues using self-statements such as “I can” and “I will not give up.” As a result, they tend to establish goals and focus on successes rather than failures. According to Seligman’s (2018) PERMA model, focusing on success and accomplishment leads to flourishing and better well-being. The current findings are compatible with prior research outcomes, showing that hope is a fundamental source of strength when coping, managing distress, and growing emotionally (Snyder, 2000; Groopman, 2006; Eche et al., 2020).

### Parents’ environmental resource: social support

Social support has been found to be the main resource helping people cope. Diener and Oishi (2005) presented social support as the most important resource that may help people cope with stress, on the one hand, and increase well-being, on the other hand. Cohen et al. (2000) pointed to the importance of social support during circumstances of illness. Thus, it is logical to expect the link between one’s support and a more positive perception of the situation to result in positive emotions.

Like the hope variable, social support was also found to be a crucial component for parents dealing with their child’s life-threatening illness. The current findings showed that parents’ greater social support contributed to their higher levels of hope, positivity ratio, and life satisfaction, as well as less negative perceptions about the impact of their child’s illness on their lives. Most research on social support resources has revealed the positive contribution of this resource on well-being (Harper et al., 2019; Gise and Cohen, 2022). Research conducted among 330 parents of children with cancer in Mexico City demonstrated a positive correlation between parents’ social support and psychological well-being (*r* = 0.25, *p* < 0.01; Toledano-Toledano et al., 2021). This important role of social support appears to coincide with Hobfoll’s (2004) conservation of resources theory, suggesting that parents who have lost resources due to their child’s illness may consciously or unconsciously seek social support to maintain their subjective well-being. Social support may also be better achieved by helping parents attain social skills, participate in parental supervision and support groups, and help them determine who in their environment could help them cope. Future research should undertake a more sensitive examination of the kinds of social support that parents need when coping with an ill child in light of Cohen et al.’s (1985) differentiation between emotional support, informational support, material support, and so on.

### Research limitations

Several limitations of the study should be mentioned. The current sample size, non-randomized recruitment method, and lack of a control group may limit the generalizability of the research findings to similar populations. A convenience sample was utilized due to the rarity of pediatric cancer in the 7-18-year-old age cohort. Furthermore, although we encountered impressively high return rates from two major medical centers (86%), some families declined to participate due to the discomfort of self-disclosing their experiences. Due to the large variety of diagnoses and the small size of each specific sickness, the study included parents of children who had been in various phases of treatment, starting from six months from the diagnosis to one year from the end of treatments. Despite the differences in the treatment phases, the research findings showed no differences between the phase groups, so we treated them as one group.

We recommend future longitudinal research to follow up on changes in subjective well-being, hope, and social support among parents of children with cancer over time, until several years after the conclusion of treatments. Follow-up research could also examine how positive personal and environmental resources may help these families adjust more effectively in transitioning back to normative life.

In addition, further empirical investigations should be conducted regarding possible intergenerational relations between parents’ and children’s well-being in the pediatric cancer context. Previous research has shown that parents’ emotional responses to stressful situations correlate with their children’s emotional responses. For example, Arabiat et al. (2018) found that a mother’s emotional strain was clearly associated with a higher risk for depression and anxiety in her child. In addition, prior research has revealed that the higher emotional and cognitive well-being of parents may impact not only their own lives but also their ability to help their children during their distressing illness (Hildenbrand et al., 2011). Research in a similar field, conducted in Germany among parents with cancer who have children under the age of 18, examined the main factors that help families develop resilience during a crisis. The findings revealed that family resilience in crisis is influenced, among other things, by social resources and open communication between family members (Heuser et al., 2024). Thus, maintaining parental well-being may have a positive effect on a child’s emotions and life satisfaction despite the pain, fears, and disruption to routine that the illness elicits (Harper et al., 2019). These possible dyadic intergenerational mechanisms should be examined in further research.

### Practical implications

This study highlights the crucial role of hope and social support as valuable resources for individuals and how perceptions are interconnected with these resources, ultimately influencing one’s overall well-being. Recognizing the significance of perception on emotional experiences, the care team should explore innovative ways to positively impact how individuals perceive their circumstances. While the diagnosis itself cannot be altered, changing the way it is perceived can significantly enhance coping strategies and adaptation to the situation, leading to improved coping and well-being.

To implement a proactive intervention, engaging with parents right from the time of diagnosis is recommended. This early engagement allows parents to express their perceptions, discuss their strengths, and identify available and lacking resources. Based on this understanding, a tailored coping intervention plan can be devised, guiding them toward accessing the necessary resources and support systems. For instance, facilitating opportunities for knowledge enhancement or connecting them with other families who have successfully coped with similar situations can be beneficial. Currently, in Israel, individualized support is provided to parents from the moment of diagnosis, continuing throughout treatment, and is administered by a multidisciplinary team specializing in crisis and trauma. Additionally, a social worker serves as the case manager, leading emotional care for both the parent and child.

In addition to the above measures, various proven tools can be employed to enhance resilience and shift perceptions. Conducting mindfulness workshops, guiding imagery exercises, introducing yoga, and incorporating other evidence-based practices are effective methods to promote positive change in perception and bolster emotional resilience. By focusing on hope, social support, and perception, and by implementing these proactive strategies, the care team can make a meaningful impact on individuals’ lives, facilitating better adaptation and improved well-being despite the challenges posed by their conditions. For example, based on the current study’s findings and with the aim of providing additional support to parents, the authors developed a family card game designed to facilitate conversations and emotional expression and support resources for both parents and children. This game aims to help families during the treatment period and beyond. It has been distributed across all oncology departments in Israel and has received positive feedback from psychologists, parents and children, indicating that it provides emotional relief on both a personal and family level.

## Conclusion

The current results suggest the importance of caring for parents along with caring for their ill child, specifically with an emphasis on understanding how parents perceive their child’s illness. The healthcare team, which accompanies parents from the moment of the child’s diagnosis, is in an excellent position to help parents achieve or maintain well-being. Creating open communication channels and using instruments to assess parents’ perceptions, hope, social support, and well-being throughout and after the treatment period, along with targeted parent-directed psychosocial interventions, when necessary, may contribute to more effective coping mechanisms in the short- and long-term (Eche et al., 2020).

## Data availability statement

The original contributions presented in the study are included in the article/supplementary material, further inquiries can be directed to the corresponding author.

## Ethics statement

The studies involving human participants were reviewed and approved by the Institutional/Ethics Review Board at the Rabin Medical Center and Tel Aviv Surasky Medical Center. The patients/participants provided their written informed consent to participate in this study.

## Author contributions

All authors listed have made a substantial, direct, and intellectual contribution to the work and approved it for publication.
